# Prediction of Noninvasive Ventilation Failure in COVID-19 Patients: When Shall We Stop?

**DOI:** 10.7759/cureus.30599

**Published:** 2022-10-23

**Authors:** Luís Neves da Silva, Rui Domingues Fernandes, Ricardo Costa, Ana Oliveira, Ana Sá, Ana Mosca, Bárbara Oliveira, Marta Braga, Marta Mendes, Alexandre Carvalho, Pedro Moreira, André Santa Cruz

**Affiliations:** 1 Department of Internal Medicine, Hospital de Braga, Braga, PRT; 2 Life and Health Sciences Research Institute (ICVS), University of Minho, Braga, PRT; 3 ICVS/3B’s—PT Government Associate Laboratory, University of Minho, Guimarães, PRT; 4 Psychological Neuroscience Laboratory, Psychology Research Center (CIPsi) School of Psychology, University of Minho, Braga, PRT

**Keywords:** resource management, prediction tools, pandemics, treatment failure, respiratory insufficiency, noninvasive mechanical ventilation, covid-19 retro

## Abstract

Introduction: In coronavirus disease 2019 (COVID-19), there are no tools available for the difficult task of recognizing which patients do not benefit from maintaining respiratory support, such as noninvasive ventilation (NIV). Identifying treatment failure is crucial to provide the best possible care and optimizing resources. Therefore, this study aimed to build a model that predicts NIV failure in patients who did not progress to invasive mechanical ventilation (IMV).

Methods: This retrospective observational study included critical COVID-19 patients treated with NIV who did not progress to IMV. Patients were admitted to a Portuguese tertiary hospital between October 1, 2020, and March 31, 2021. The outcome of interest was NIV failure, defined as COVID-19-related in-hospital death. A binary logistic regression was performed, where the outcome (mortality) was the dependent variable. Using the independent variables of the logistic regression a decision-tree classification model was implemented.

Results: The study sample, composed of 103 patients, had a mean age of 66.3 years (SD=14.9), of which 38.8% (40 patients) were female. Most patients (82.5%) were autonomous for basic activities of daily living. The prediction model was statistically significant with an area under the curve of 0.994 and a precision of 0.950. Higher age, a higher number of days with increases in the fraction of inspired oxygen (FiO_2_), a higher number of days of maximum expiratory positive airway pressure, a lower number of days on NIV, and a lower number of days from disease onset to hospital admission were, with statistical significance, associated with increased odds of death. A decision-tree classification model was then obtained to achieve the best combination of variables to predict the outcome of interest.

Conclusions: This study presents a model to predict death in COVID-19 patients treated with NIV in patients who did not progress to IMV, based on easily applicable variables that mainly reflect patients’ evolution during hospitalization. Along with the decision-tree classification model, these original findings may help clinicians define the best therapeutical approach to each patient, prioritizing life-comforting measures when adequate, and optimizing resources, which is crucial within limited or overloaded healthcare systems. Further research is needed on this subject of treatment failure, not only to understand if these results are reproducible but also, in a broader sense, helping to fill this gap in modern medicine guidelines.

## Introduction

Coronavirus disease 2019 (COVID-19) has affected millions of people worldwide [1]. Severe acute respiratory syndrome coronavirus 2 (SARS-CoV-2) may still spread exponentially, especially when preventive measures are not fully adopted. Although most infected individuals do not require hospitalization [2], we continue to observe a large group of patients who need advanced respiratory support [3]. The dramatic lack of ventilators felt worldwide during the COVID-19 pandemic confirmed that, in a setting of limited or overloaded healthcare systems, optimizing the use of medical resources is paramount [4,5].

In the first months of the pandemic, the idea of early intubation was widespread, based on previous experience with acute respiratory distress syndrome (ARDS) [6,7], which resulted in overstrained intensive care units (ICU). Over time, other forms of respiratory support that were more available and less demanding than invasive mechanical ventilation (IMV), such as high-flow nasal oxygen (HFNO) and noninvasive ventilation (NIV), became advocated [8,9].

NIV efficacy is clearly validated in various diseases that cause respiratory failure, such as cardiogenic pulmonary edema or exacerbation of chronic obstructive pulmonary disease (COPD) [10]. Although controversial, the use of NIV has also been supported in cases of ARDS and community-acquired pneumonia, by improving clinically relevant outcomes such as progression to intubation [11,12].

The use of NIV in COVID-19 was initially limited by a lack of information regarding its benefit and concerns about aerosolization [13]. However, when it started to be used due to necessity and lack of better alternatives, studies showed an improvement in mortality rates, hospitalization time, and a decrease in the severity of symptoms related to COVID-19 when comparing NIV against IMV [14]. Moreover, no difference in mortality was found between patients who started NIV and later progressed to IMV and those who started on IMV [9]. A complementary study found that patients who commenced continuous positive airway pressure (CPAP) therapy earlier had lower mortality than patients commencing CPAP later [15]. As more research emerged, the usage of helmet NIV instead of HFNO was linked to a reduction in the number of patients who needed IMV [16].

Due to the success of those strategies, a sequential model of care (conventional oxygen, HFNO, NIV, and finally IMV) became recommended and standardized [17]. Optimal criteria to step up, however, remained difficult to accomplish in most hospitals due to the high influx of patients, and lack of equipment or personnel. It became mandatory to allocate resources to patients that could benefit the most of them, meaning limitation of care in certain cases and recognition of treatment failure in others, especially in those not candidates for IMV. Very few studies have addressed the risk of NIV failure in COVID-19 and those that had, mainly focus on analytical data, comorbidities, age, and some clinical parameters [18-24]. To our knowledge, no work postulates a model to predict NIV failure in COVID-19, which includes the evolution of NIV parameters, such as the fraction of inspired oxygen (FiO_2_) and end positive airway pressure (EPAP), which could help guide clinical decisions.

In modern medicine, despite the high frequency of treatment failure in many scenarios, current clinical guidelines are mainly oriented toward adding treatments and rarely provide support to decisions involving the suspension of a therapy that has apparently become futile in a certain patient. Sadly, that is also happening in COVID-19, where studies and orientations on when to change focus and reinforce life-comforting measures are scarce [25,26]. As sensible practitioners, we need to keep in mind that the urge to prolong a patient's life may negatively impact its quality, particularly in the last moments of it. Insisting, perhaps obsessively, on NIV when the patient will ultimately die, will lead to dysthanasia and, in a limited resource setting, preclude other patients from receiving beneficial therapies.

The main purpose of this study is to elaborate a model that, in a reliable and timely manner, predicts NIV failure in patients who did not progress to IMV. These results may help to assess the futility of therapy, avoiding life-prolonging measures of no benefit to the patient. After all, we were taught that first, we shall do no harm.

This article was previously posted on the Research Square preprint server on February 9, 2022 [27].

## Materials and methods

Study design and setting

This is a retrospective observational study with COVID-19 patients admitted to a Portuguese tertiary hospital, Hospital of Braga, Braga, Portugal, between October 1, 2020, and March 31, 2021. The hospital had an inpatient capacity of 130 beds for COVID-19 patients, of which 32 were ICU beds and 16 had continuous monitoring. In this study, baseline characteristics and differences in the clinical evolution of two different groups of patients (dead/alive) treated with NIV were compared. This study was approved by the Ethical Committee for Health of the Hospital of Braga (reference 61_2021).

Participants’ selection and formation of groups for analysis

The medical records of all patients hospitalized for COVID-19 during the study period were consulted to exclusively select patients undergoing NIV during hospitalization. Subsequently, compliance with inclusion and exclusion criteria was verified for each case.

Inclusion criteria were as follows: age ≥18 years; confirmed COVID-19 diagnosis based on symptoms and positive polymerase chain reaction (PCR) test; COVID-19-related acute respiratory insufficiency; need of NIV either as bilevel positive airway pressure (BPAP) or CPAP for more than 24 hours; initiation of the ventilatory support before the 20^th ^day of symptoms; use of the ventilators Philips Respironics V60, Philips Respironics V60 Plus, or Philips Respironics Trilogy Evo (Koninklijke Philips N.V., Amsterdam, Netherlands).

Exclusion criteria were as follows: treatment with IMV during hospitalization; active hematological malignancy; active stage III/IV cancer; ongoing chemotherapy; chronic use of NIV for other respiratory conditions, without any change in the usual therapy mode or parameters; death during hospital stay not attributable to COVID-19; hospital admission due to conditions not related to COVID-19.

Patients treated with IMV were excluded so that the group of surviving patients was unequivocally a therapeutically successful group of NIV and not of other supplementary strategies. Likewise, the group of patients who died, as they were not treated with IMV, unequivocally corresponds to NIV failure and not to the concomitant failure of NIV and IMV.

Participants’ data collection

Patients’ medical records were used to obtain baseline data on demographic features and comorbidities (age, sex, functional and cognitive status, tabagism and alcohol abuse, obesity, arterial hypertension, diabetes mellitus, dyslipidemia, heart failure, chronic liver disease, chronic kidney disease, previous stroke, immune-mediated diseases, active cancer (stages I and II), COPD, asthma, pulmonary embolism, pulmonary hypertension, other respiratory diseases, previous long term oxygen therapy), as well as information regarding the course of the disease and the use of NIV during the hospital stay. Details on CPAP and BPAP modes, namely the values of EPAP and inspiratory positive airway pressure (IPAP), as well as FiO_2_, were collected from the time the patient started NIV until the last day of its utilization. More specifically, for each 24-hour period of NIV, the maximum CPAP/ EPAP value (referred now on as EPAP for simplification) was collected as well as the maximum FiO_2_ and IPAP used with that EPAP value. All data were collected and inserted into a database by the medical doctors involved in this work.

Outcome measure

The outcome measure of interest was NIV failure, defined as COVID-19-related in-hospital death.

Statistical analysis

Descriptive statistics were obtained for sociodemographic and clinical variables. The assumption of normality was assessed with the Shapiro-Wilk test. Sociodemographic features with a prevalence higher than 10% and clinical variables were compared between groups of patients (alive vs dead) using the independent samples t-test, the Mann-Whitney, or the chi-square (X^2^) test in the case of categorical variables.

Data were analyzed with a binary logistic regression model, where the outcome (0 - alive; 1 - dead) was set as the dependent variable. The predictors of interest included age; sex; functional status; the number of days from disease onset to hospital admission (when the onset of symptoms was unknown, date of diagnosis was considered), and NIV features for each patient during hospital stay: number of days on NIV; average values of EPAP and FiO_2_; maximum values of EPAP and FiO_2_; the number of days with the maximum EPAP. To reflect the evolution of NIV features, two variables were created: the number of days with an increase in EPAP and the number of days with an increase in FiO_2_. The area under the curve (AUC), sensitivity, specificity, and precision were extracted as performance metrics of this model.

A decision-tree classification model was implemented to further explore the combination of variables that best predicts the outcome of interest. To achieve this, the same variables defined as independent variables in the logistic regression model were entered as features of interest. With the decision-tree classification model, we aim to divide the dataset of alive and non-alive patients into homogeneous groups, based on successive decision boundaries established according to relevant features that minimize the probability of a random element being incorrectly classified (the Gini impurity).

Descriptive statistics and logistic regression analysis were performed using the free and open-source computer software JASP (Jeffreys's Amazing Statistics Program) (Version 0.16). The decision-tree classification model was implemented with scikit-learn (Version 1.0.1) [28]. Statistical significance was defined at the p*<.05* level.

## Results

Patient characteristics at hospital admission and group differences

**Figure 1:**
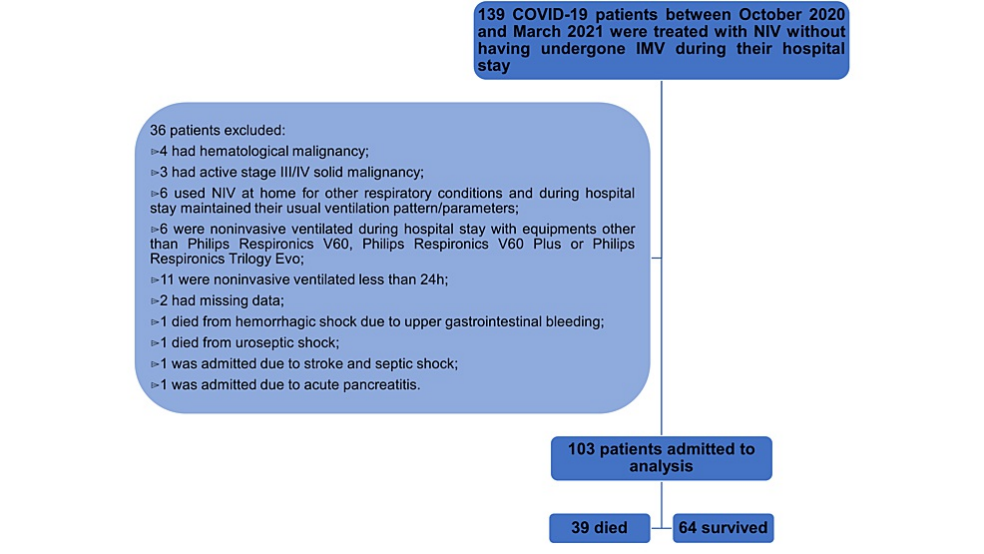
Flowchart of inclusion and exclusion of participants Between October 1, 2020, and March 31, 2021, 139 COVID-19 patients, were treated with noninvasive ventilation. These patients were not treated with invasive mechanical ventilation at any point during their hospital stay. Applying exclusion criteria, 103 patients were admitted to analysis, of which 39 died during the hospital stay. COVID-19: coronavirus disease 2019; NIV: noninvasive ventilation; IMV: invasive mechanical ventilation

One hundred thirty-nine patients treated with NIV between October 1, 2020, and March 31, 2021, were identified, of which 103 met the inclusion and exclusion criteria and were eligible for analysis. Of these, 39 patients (37.9%) died during their hospital stay due to COVID-19 (Figure 1).

The mean age was 66.3 years (SD=14.9), and 40 patients (38.8%) were women. No patient was vaccinated against COVID-19. Patients who died were significantly older. Arterial hypertension was the most common comorbidity (71.8%) followed by dyslipidemia (52.4%), obesity (35.9%), and diabetes mellitus (31.1%). Eighty-five patients (82.5%) were autonomous for daily activities. Patients with arterial hypertension, dyslipidemia, heart failure, a compromised cognitive status, and those who were non-autonomous, were more likely to die. Patients presented to the hospital from disease onset in a median time of six days (IQR=3.5-8.0). The median time to presentation was significantly lower in patients who died compared to those who remained alive, as well as the length of stay in the hospital. More detailed information on the population’s characteristics at hospital admission is shown in Table 1.

**Table 1:** Patient characteristics and group differences COPD, chronic obstructive pulmonary disease; CKD, chronic kidney disease; CLD, chronic liver disease; FIO_2_, fraction of inspired oxygen; HF, heart failure; LTOT, long-term oxygen therapy; No., numbers of patients included in the analysis; PaO_2_, partial pressure of oxygen in the arterial blood; PE, pulmonary embolism; PH, pulmonary hypertension.

Characteristic	Total (n=103)	Dead (n=39)	Alive (n=64)	Group comparison
Age, mean (SD), y	66.3 (14.9)	76.1 (10.8)	60.4 (14.0)	t(101)=-6.011; p<0.001
Sex, No. (%)				
Female	40 (38.8%)	17 (43.6%)	23 (35.9%)	X^2^=0.597; p=0.44
Male	63 (61.2%)	22 (56.4%)	41 (64.1%)	
Functional Status, No. (%)				
Autonomous	85 (82.5%)	25 (64.1%)	60 (93.7%)	X^2^=14.769; p<0.001
Non-autonomous	18 (17.5%)	14 (35.9%)	4 (6.3%)	
Cognitive Status, No. (%)				
Preserved	94 (91.3%)	32 (82.1%)	62 (96.9%)	X^2^=6.678; p=0.01
Compromised	9 (8.7%)	7 (17.9%)	2 (3.1%)	
Obesity, No. (%)	37 (35.9%)	11 (28.2%)	26 (40.6%)	X^2^=1.624; p=0.20
Arterial Hypertension, No. (%)	74 (71.8%)	33 (84.6%)	41 (64.1%)	X^2^=5.061; p=0.02
Diabetes Mellitus, No. (%)	32 (31.1%)	14 (35.9%)	18 (28.1%)	X^2^=0.684; p=0.41
Dyslipidemia, No. (%)	54 (52.4%)	27 (69.2%)	27 (42.2%)	X^2^=7.106; p=0.008
Tabagism, No. (%)				
Previous	7 (6.8%)	3 (7.7%)	4 (6.3%)	
Current	3 (2.9%)	1 (2.6%)	2 (3.1%)	
Alcohol abuse, No. (%)				
Previous	6 (5.8%)	3 (7.7%)	3 (4.7%)	
Current	5 (4.9%)	2 (5.1%)	3 (4.7%)	
Immune-mediated diseases, No. (%)				
Still's disease	1 (1.0%)	0 (0.0%)	1 (1.6%)	
Rheumatoid Arthritis	1 (1.0%)	0 (0.0%)	1 (1.6%)	
Psoriasis	1 (1.0%)	1 (2.6%)	0 (0.0%)	
Inflammatory Bowel Disease	1 (1.0%)	0 (0.0%)	1 (1.6%)	
Immune Thrombocytopenia	1 (1.0%)	1 (2.6%)	0 (0.0%)	
Stage I/II Active Cancer, No. (%)	0 (0.0%)	0 (0.0%)	0 (0.0%)	
COPD, No. (%)	7 (6.8%)	3 (7.7%)	4 (6.3%)	
Asthma, No. (%)	3 (2.9%)	1 (2.6%)	2 (3.1%)	
Obstructive Sleep Apnea, No. (%)	9 (8.7%)	4 (10.3%)	5 (7.8%)	
Hypersensitivity Pneumonitis, No. (%)	1 (1.0%)	1 (2.6%)	0 (0.0%)	
PH, No. (%)	1 (1.0%)	1 (2.6%)	0 (0.0%)	
Previous PE, No. (%)	0 (0.0%)	0 (0.0%)	0 (0.0%)	
LTOT, No. (%)	1 (1.0%)	0 (0.0%)	1 (1.6%)	
HF, No. (%)	17 (16.5%)	14 (35.9%)	5 (7.8%)	X^2^=12.706; p<0.001
Previous Stroke, No. (%)	6 (5.6%)	6 (15.4%)	0 (0.0%)	
CLD, No. (%)				
Child A	2 (1.9%)	1 (2.6%)	1 (1.6%)	
Child B	0 (0.0%)	0 (0.0%)	0 (0.0%)	
Child C	0 (0.0%)	0 (0.0%)	0 (0.0%)	
CKD, No. (%)				
G3	7 (6.8%)	5 (12.8%)	2 (3.1%)	
G4	0 (0.0%)	0 (0.0%)	0 (0.0%)	
G5	2 (1.9%)	2 (5.1%)	0 (0.0%)	
PaO_2_/FiO_2_ ratio at admission, median (IQR)	204.0 (114.0-247.3)	212.0 (131.5-254.0)	195.5 (108.4-247.2)	U(101)=1180; p=0.65
Days from disease onset to hospital admission, median (IQR)	6.0 (3.5-8.0)	4.0 (0.0-6.0)	7.0 (5.0-9.0)	U(101)=1852; p<0.001
Length of stay, median (IQR)	13.0 (9.0-21.3)	9.0 (6.0-12.0)	17.0 (13.0-26.0)	U(101)=1942.5; p<0.001

NIV features and evolution during the hospital stay

The median time on NIV was six days (IQR=4.0-9.0 days) without a significant difference between patients who died or survived. Patients who died had significantly higher average values of EPAP, maximum EPAP, number of days on maximum EPAP, average values of FiO_2_, and maximum FiO_2_. The number of days with increases in EPAP was also significantly higher in those who died as well as the number of days with increases in FiO_2_. These and other results are detailed in Table 2. 

**Table 2:** Group differences regarding NIV features-related variables EPAP, end positive airway pressure; FIO_2_, fraction of inspired oxygen; HFNO, high-flow nasal oxygen; NIV, noninvasive ventilation; IQR, interquartile range

Characteristics	Total (n=103)	Dead (n=39)	Alive (n=64)	Group comparison
Days on NIV, median (IQR) (min-max)	6.0 (4.0-9.0) (2-28)	7.0 (4.5-9.0) (2-23)	6.0 (4.0-9.3) (2-28)	U(101)=1174.5; p=0.62
Average EPAP, median (IQR)	10.2 (9.5-11.2)	11.0 (10.3-12.0)	10.0 (9.1-10.5)	U(101)=633.0; p<0.001
Maximum EPAP, median (IQR)	12.0 (10.0-12.0)	12.0 (12.0-12.0)	12.0 (10.0-12.0)	U(101)=781.0; p<0.001
Days with maximum EPAP, median (IQR)	1.0 (1.0-2.0)	2.0 (1.0-2.0)	1.0 (1.0-1.0)	U(101)=857.5; p=0.002
Average FiO_2_, median (IQR)	68.3 (62.6-81.2)	85.0 (77.5-91.5)	65.0 (60.6-68.6)	U(101)=344.5; p<0.001
Maximum FiO_2_, median (IQR)	80.0 (70.0-100.0)	100.0 (90.0-100.0)	75.0 (68.8-81.3)	U(101)=489.5; p<0.001
Days with conventional oxygen before NIV, median (IQR)	1.0 (0.0-2.0)	1.0 (0.0-2.0)	0.0 (0.0-2.0)	U(101)=1114.0; p=0.33
Days with HFNO before NIV, median (IQR)	0.0 (0.0-1.0)	0.0 (0.0-1.0)	0.0 (0.0-1.0)	U(101)=1275.0; p=0.84
Days with EPAP increases, median (IQR)	1.0 (0.0-1.5)	1.0 (0.0-2.0)	0.0 (0.0-1.0)	U(101)=879.5; p=0.007
Days with FiO_2_ increases, median (IQR)	1.0 (0.0-3.0)	1.0 (1.0-3.0)	1.0 (0.0-2.0)	U(101)=857.5; p=0.005

Logistic regression model

Due to the high correlation between average FiO_2_ and maximum FiO_2_ values (r(101)=0.713; *p<.001*) as well as average EPAP and maximum EPAP (r(101)=0.807; *p<.001*), only the average values of EPAP and FiO_2_ were retained in the model. As the main purpose was to build a model based on NIV features, comorbidities that were prevalent and associated with death in our sample (arterial hypertension, dyslipidemia, and heart failure) were not included, also serving to lessen the risk of overfitting the model.

**Figure 2:**
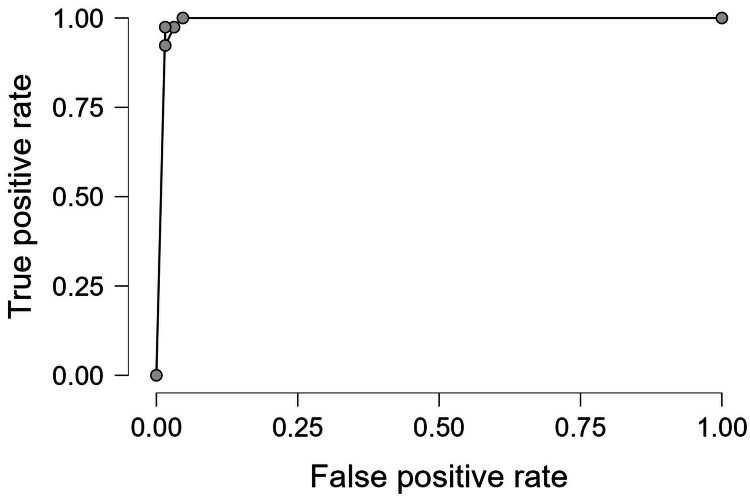
Receiver operating characteristic curve for the predictive model The receiver operating characteristic curve for the predictive model has an area under the curve of 0.994 and a precision of 0.950.

The logistic regression model was statistically significant (X^2^=119.865; *p<.001*) with an AUC of 0.994, a sensitivity of 0.974, a specificity of 0.969, and a precision of 0.950. The model’s receiver operating characteristic (ROC) curve is shown in Figure 2.

Increased age, a higher number of days with increases on FiO_2_, a lower number of days on NIV, a higher number of days with EPAP at maximum values, and a lower number of days from disease onset to hospital admission were associated with increased odds of death (Table 3).

**Table 3:** Binary logistic regression EPAP, end positive airway pressure; FIO_2_, fraction of inspired oxygen; NIV, noninvasive ventilation

					Wald Test		95% Confidence interval	
	Estimate	Standard Error	Odds Ratio	Z	Wald Statistic	p	Lower bound	Upper bound
(Intercept)	-40.827	15.975	1.858e-18	-2.556	6.531	0.01	-72.138	-9.516
Sex	2.090	1.653	8.083	1.264	1.598	0.21	-1.150	5.329
Age	0.288	0.110	1.334	2.629	6.910	0.009	0.073	0.503
Functional Status	3.162	1.967	23.617	1.607	2.584	0.11	-0.693	7.017
Days with FiO_2_ increases	4.789	1.705	120.176	2.809	7.890	0.005	1.447	8.131
Average EPAP	1.307	0.884	3.696	1.478	2.186	0.14	-0.426	3.040
Average FiO_2_	0.131	0.071	1.140	1.836	3.370	0.07	-0.009	0.270
Days on NIV	-1.713	0.628	0.180	-2.727	7.435	0.006	-2.944	-0.482
Days with maximum EPAP	3.511	1.566	33.469	2.242	5.027	0.03	0.442	6.580
Days from disease onset to hospital admission	-0.839	0.362	0.432	-2.316	5.364	0.02	-1.549	-0.129

Decision-tree classification model

**Figure 3:**
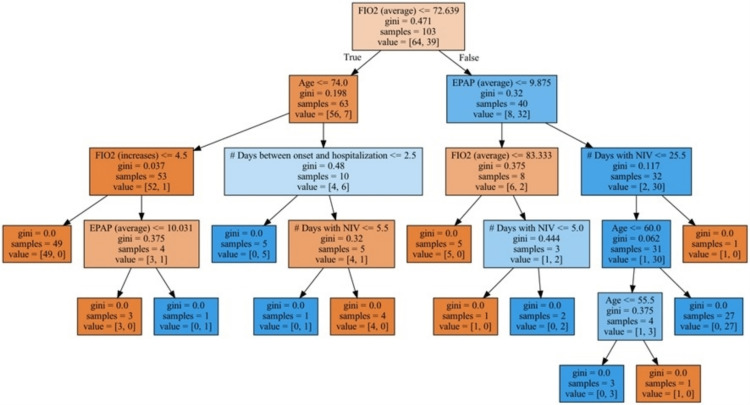
Decision-tree classification model The decision-tree classification model presented starts with a root node in which all the observations (n_total_=103; n_alive_=64; n_dead_=39) are present. The first decision boundary was based on the average fraction of inspired oxygen (FiO_2_) values: approximately 82% of the patients who die have average FiO_2_ values greater than 72.6%. A new decision boundary is defined by age in the group of patients with average FiO_2_ values equal to or lower than 72.6%. Of a total of seven patients in this branch, six patients with ages greater than 74 years die. Among the group of patients with average FiO_2_ values greater than 72.6%, from a total of 32 patients, 30 patients with end positive airway pressure (EPAP) with average values greater than 9.9 die. Third-level decision boundaries are established according to FiO_2_ average values, number of days with increases in FiO2, number of days between disease onset and hospitalization, and the total number of days on noninvasive ventilation (NIV). The word "gini" that is present in the figure refers to the Gini coefficient, which ranges from 0 to 1.

The model represented in Figure 3 starts with a root node in which all the observations (n_total_=103; n_alive_=64; n_dead_=39) are present. The first decision boundary was based on the average FiO_2_ values: approximately 82% of the patients who die have average FiO_2_ values greater than 72.6%. A new decision boundary is defined by age in the group of patients with average FiO_2_ values equal to or lower than 72.6%. Of a total of seven patients in this branch, six patients with ages greater than 74 years die. Among the group of patients with average FiO_2_ values greater than 72.6%, from a total of 32 patients, 30 patients with EPAP average values greater than 9.9 die. Third-level decision boundaries are established according to FiO_2_ average values, the number of days with increases in FiO_2_, the number of days between disease onset and hospitalization, and the total number of days on NIV.

## Discussion

A mortality rate of 37.9% was observed in the sample of this study. Patients had a mean age of 66.3 years and 61.2% were males. Arterial hypertension was the most common comorbidity (71.8%) and there was a high prevalence of other cardiometabolic comorbidities. Overall, the mortality and patient characteristics are in accordance with other studies of COVID-19 patients treated with NIV [29].

It should come as no surprise that patients who died were significantly older and had a worse functional status [30,31]. These two factors alone can be easily determined on admission and provide a basis for predicting patient response to NIV. Patients who died had a lower number of days on NIV, which may seem surprising at first sight. Revising our data, 71% of those who died were treated for a period of fewer than five days and 50% of patients of them were older than 70 years. Moreover, patients who died had significant differences in several variables that reflect the need for greater ventilatory support: higher average FiO_2_ and EPAP, higher maximum FiO_2_ and EPAP as well as a higher number of days with increases in FiO_2_ and EPAP, and a higher number of days with EPAP on maximum values. This may reflect a rapidly progressive disease course in a large group of patients. Survivors perhaps had a slower progression of the disease, were able to control inflammation, and reach a plateau before their condition started to improve. In COVID-19, the recovery process is usually slow, potentially justifying why survivors had more days under NIV. This explanation also elucidates why patients who died had significantly less time from disease onset to hospital admission, that is, it may reflect a more aggressive inflammatory response, marking a worse clinical trajectory, as has been suggested [32].

A logistic regression model, which was statistically significant with an area under the curve of 0.994, a sensitivity of 0.974, a specificity of 0.969, and a precision of 0.950, was obtained. Based on the variables used in the logistic regression model, a decision tree was developed, which allows the medical team to quickly stratify a patient into a group of similar patients and extrapolate mortality rates. For instance, it is striking that mortality of 93.8% is expected in a group of patients with an average FiO_2_ greater than 72.6% and an average EPAP greater than 9.9. We can further stratify the risk by continuing down the decision tree, but such a high mortality rate will necessarily promote a clinical discussion about the patient’s best interest and the most adequate therapeutic approach.

Clinicians have progressively more data and tools available to predict a patient's prognosis. For example, Liu et al. have found several parameters in their study that also help predict NIV failure and were able to develop an online calculator to assist decision-making [24]. Works like this have been mainly limited to predicting NIV failure based on analytical parameters and comorbidities. While these kinds of predictors are undoubtedly important, our model was intentionally built upon variables that reflect the evolution throughout the hospital stay, making this work innovative, useful to clinical practice, and helpful in filling a gap in the literature. Overall, works of this kind may become crucial in settings of scarce medical resources, be it from a chronic lack of resources or from unexpected demand. Accurate assessment of a patient's prognosis allows for better employment of resources, treating those who may benefit from the continuation of advanced therapeutic measures, and giving more thought and time to life-comforting measures which are often regrettably omitted. It must be stated, however, that the results provided should not override clinical sense or wisdom.

Limitations

This study has a retrospective observational design, which does not allow to rule out residual confounding, and has an inferior level of evidence. Data were collected from medical records, which are prone to human error and are not always complete or updated. Most patients initiated NIV when HFNO failed to maintain a partial pressure of oxygen (PaO_2_)>60 mmHg with a FiO_2_>70%. Progression to IMV was based on clinical judgment, case by case, and was not always consistent throughout the pandemic, which may have interfered with sample selection. All patients received the standard of care with dexamethasone, with none receiving other agents such as remdesivir, tocilizumab, or antibiotics. These practices can differ from other centers, making it hard to extrapolate our findings. Similarly, centers with access to distinct models of ventilators that perform differently may experience non-identical results. We may also hypothesize that a sample of fully vaccinated individuals could have shown different mortality rates and patterns. Finally, the number of patients in our center made it impossible to have a separate cohort for cross-validation of the model, a technique to lessen the risk of overfitting, which ideally is done with samples from other centers. 

## Conclusions

This retrospective observational study, despite its limitations, presents a model to predict NIV failure, defined as COVID-19-related in-hospital death, in patients who did not progress to IMV, with an area under the curve of 0.994 and a precision of 0.950, based on easily applicable variables that also reflect evolution during hospital stay (i.e. sex; age; functional status; days from disease onset to hospital admission; days on NIV; average FiO_2_; days with FiO_2_ increases; average EPAP; days with maximum EPAP), that was further explored with the implementation of a decision-tree classification model, allowing for the best combination of variables to predict mortality. These findings add to other studies on NIV use during COVID-19 and may help clinicians define the best therapeutical approach to each patient, prioritizing life-comforting measures when adequate and optimizing resources, which is crucial within limited or overloaded healthcare systems. Further research is needed on this subject of treatment failure, not only to understand if these results are reproducible but also, in a broader sense, helping to fill this gap in modern medicine guidelines.
